# Delayed radial head dislocation after radial shaft fracture fixation: a case report and review of the literature

**DOI:** 10.1186/s12893-022-01514-1

**Published:** 2022-02-17

**Authors:** Jiyong Yang, Jie Zhang, Zhengzhong Yang

**Affiliations:** Department of Orthopaedics, Shenzhen Ping Le Orthopaedic Hospital, Shenzhen, 518000 Guangdong China

**Keywords:** Radial head dislocation, Radial fracture, Delayed, Ipsilateral

## Abstract

**Background:**

Ipsilateral fracture of the radial shaft with dislocation of the radial head was a rare injury, but a delayed radial head dislocation after radial shaft fracture fixation was more extremely rare.

**Case presentation:**

A 39-year-old man fell from the height on his outstretched hand and injured his left, non-dominant forearm. Preoperative radiographs demonstrated a comminuted fracture of the proximal third of the radius but with no apparent dislocation of the distal or proximal radioulnar joints or the elbow. Seven days after the injury, the radius was fixed with a reconstruction locking plate, and the immediate postoperative radiograph revealed a satisfactory reduction. However, a radiograph done at the 4th week postoperatively showed that the radial head dislocated. Manual reduction under anesthesia was tried but failed and the patient refused to take another open surgery. The patient had an acceptable range of motion 12 months after the surgery: elbow flexion 120°, full elbow extension, forearm pronation 80°, forearm supination 80°, but he complained the pain around the elbow.

**Conclusion:**

In the case of radial shaft fracture especially the when occurs at the proximal third of the radial shaft, even if the radiograph does not show the injury of the proximal radioulnar joint, we should also make a thorough examination of the proximal radioulnar joint. If the radial head dislocation is not initially diagnosed or treated late, a delayed dislocation would be very difficult to manage with a poor expected outcome.

## Background

Dislocation of the radial head is commonly associated with fracture of the proximal ulna which is referred to as Monteggia fracture. However, a fracture of the radial shaft combined with radial head dislocation was a rare injury. This condition is scarcely described in the literatures [1–4]. In the majority of cases, the radial head dislocated at the time of injury; but a delayed dislocation of the radial head (dislocation of the proximal radioulnar joint and by necessity the radiocapitellar joint) was hardly ever mentioned and mechanism of this type of injury was unclear. Here we present a case of delayed dislocation of the radial head after open reduction and internal fixation of a fracture of the proximal third of the radial shaft.

## Case presentation

**Fig. 1:**
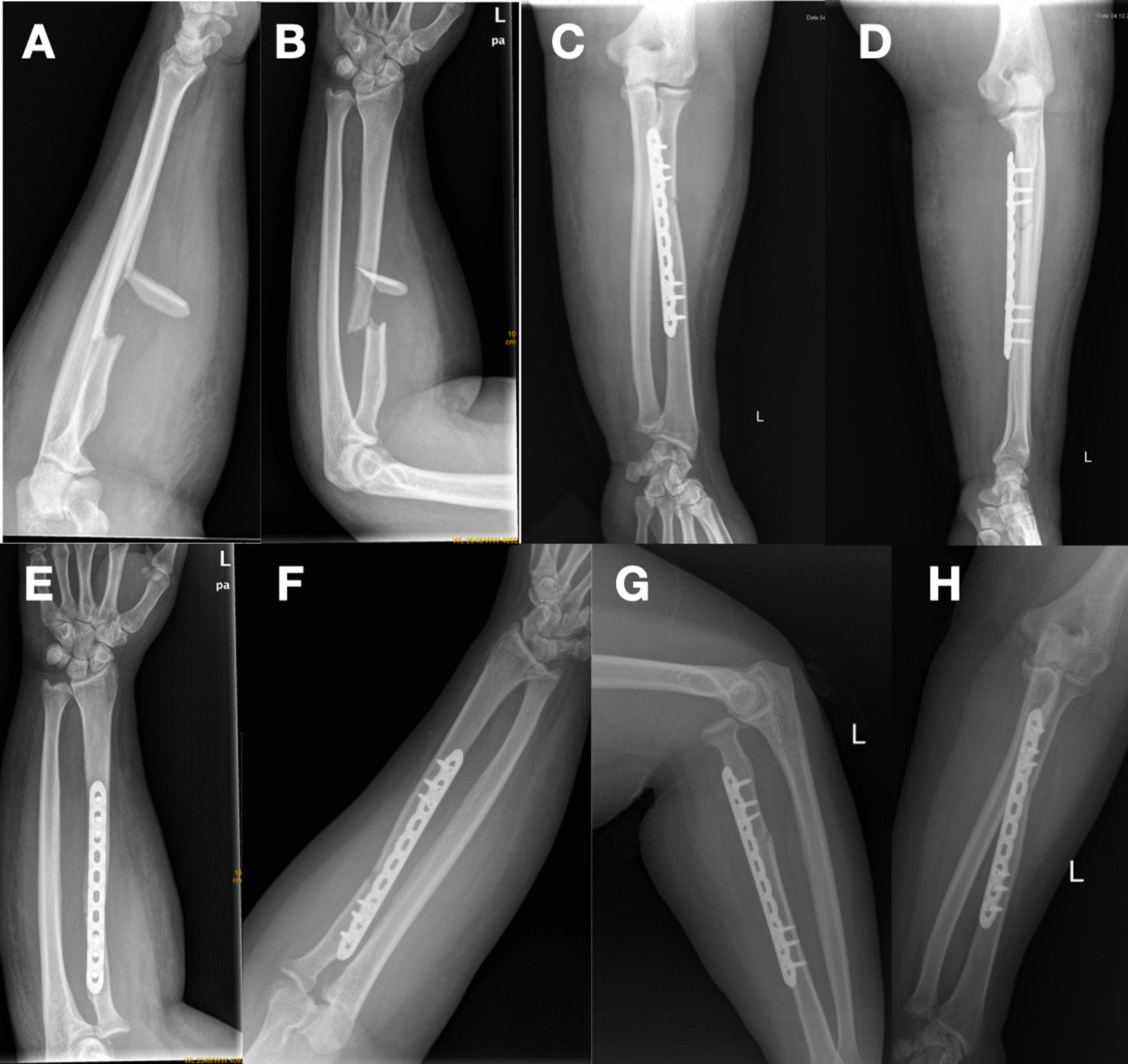
**A**, **B** Preoperative radiographs revealed a comminuted fracture of the proximal third of the radius; **C**, **D** The immediate postoperative radiograph; **E**, **F** 4th week after operation. **G**, **H** 12 months after the operation

**Fig. 2:**
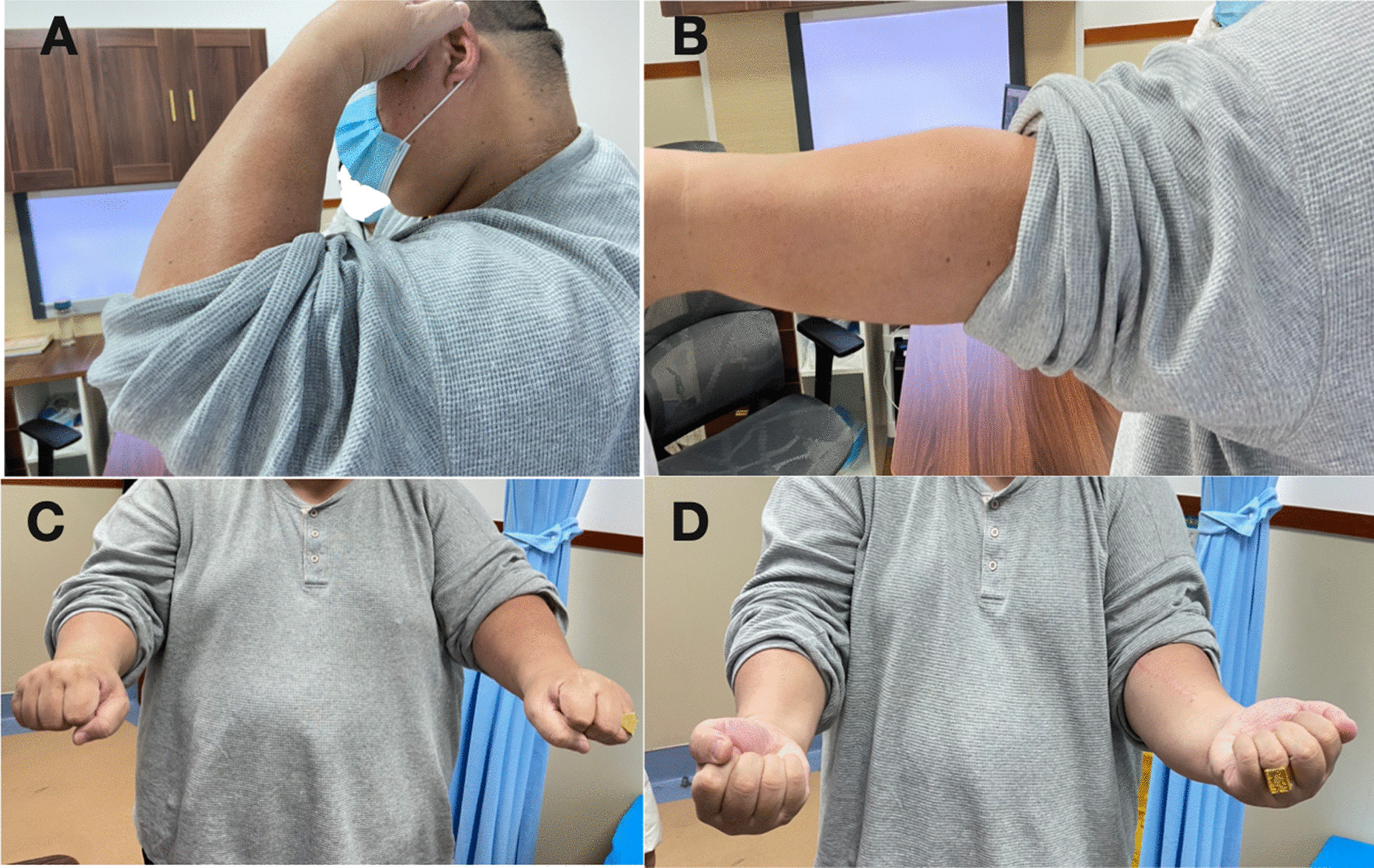
The range of motion 12 months postoperatively: **A** elbow flexion; **B** elbow extension; **C** forearm pronation; **D** forearm supination

A 39-year-old man fell from the height on his outstretched hand and injured his left, non-dominant forearm. Physical examination of the left forearm revealed generalised tenderness and swelling of the forearm. He had a limited range of movement of both the elbow and forearm, but we did not examine his elbow in detailed because he denied the injury of elbow. He had no nerve dysfunction. Radiographs demonstrated a comminuted fracture of the proximal third of the radial shaft but with no apparent dislocation of the distal or proximal radioulnar joints or the elbow (Fig. 1A, B). Seven days after the injury, we fixed the radius with a reconstruction locking plate, applied through Henry’s volar approach. The immediate postoperative radiograph revealed satisfactory reduction and stable fixation (Fig. 1C, D). Considering his high BMI index, the limb was immobilized in an adjustable brace for 4 weeks with the elbow flexed at 90° and the forearm in supination. However, from the first day after surgery, we allowed gradually active flexion and extension of elbow twice a day and no forearm rotation. However, a radiograph done at the 4th week postoperatively showed that the radial head was dislocated (Fig. 1E, F), but the patient did not complain of any particular discomfort during the recovery. We planned to perform the magnetic resonance imaging of the elbow to assess the ligaments around it and suggested the patient to take an open reduction of the proximal radioulnar joint but he refused, so we only had to try a manual reduction under anesthesia. There was no doubt that we failed, the patient opted not to pursue any additional treatment. At the time of 1 month after surgery, he currently has a restricted range of elbow flexion and forearm rotation: elbow flexion 70°, full elbow extension, forearm pronation 60°, forearm supination 20°. At the time of 1 year after surgery (Fig. 1G, H), he achieved an acceptable range of motion (Fig. 2): elbow flexion 120°, full elbow extension, forearm pronation 80°, forearm supination 80°, but he still complained the pain around the elbow, with a Disabilities of the Arm, Shoulder and Hand scored of 32.

## Discussion and conclusion

Monteggia fracture is a rare injury, which represents only 0.7% of all elbow fracture-dislocations in adult patients [5]. As for the ipsilateral radial shaft fracture with dislocation of the radial head, it was more rare, and in the majority of literature, the radial head dislocated at the time of injury [6–10]. While in our unique case, the preoperative radiograph only revealed a radial shaft fracture and the radial head dislocation was detected 4 weeks after the surgery which was even more rare.

The first case of ipsilateral radial shaft fracture with dislocation of the radial head, as far as we know, was reported by JM Simpson in 1991 [11]. Such injuries were subsequently reported in a succession of articles. Mehara et al. reported a case of anteroinferior radial head dislocation with radial shaft fracture [4]. In these cases, the radial head dislocation was diagnosed preoperatively. However, a delayed radial head dislocation was hardly ever reported. To date, there were only three cases showed a delayed dislocation with ipsilateral radial shaft fracture. Hiroshi Yamazaki et al. reported a case of delayed radial head dislocation associated with malunion of radial shaft fracture [12]. Linzel et al. reported 4 cases of of radial shaft fractures associated with dislocation of the proximal radioulnar joint [6], one of their cases showed the similar situation with us which was also noted a dislocation of the proximal radioulnar joint 1 month after repair of the radial shaft. A young adult male also showed a postoperatively dislocation of the radial head reported by Jagdeep Singh et al. [13], but this case did not have neither the preoperative nor the intra-operative radiographs which was unclear whether he had dislocation of radial head preoperatively or not. Due to the scarcity of cases, we are still lack of recognition of this kind of injury.

Annular ligament, quadrate ligament, and proximal half of the interosseous membrane are important factors in maintaining the stability of radial head. In the case of the simultaneously ipsilateral radial shaft fracture with dislocation of the radial head, most of the cases spoke of a mechanism of hyperpronation of the forearm combined with an elbow hyperflexion. However, mechanism of a delayed dislocation remains unclear, there could be several mechanisms. Firstly, the varus stress applied at the elbow would disrupte the annular ligament, and it caused dislocation of the radial head, then the subsequent violence resulting in fracture of radial shaft. Under this circumstance, it is possible that some patients would occur spontaneous relocation of the elbow joint which may lead to the misdiagnosis [6]. And through the literatures, we also noted that the fracture overwhelmingly occurred in the proximal third of the radial shaft and often combined with a butterfly fragment which may include the origin of the interosseous ligament [4, 6, 9, 13]. The failure to restore the fragment of the radius on which the interossesous ligament insert could also lead to the dislocation of the radial head postoperatively [6]. The angular or the rotational deformities of fractures can also pull the radial head out especially when the annular ligament was injured but this could be easily detected. When handling a delayed radial head dislocation, we should carefully evaluate the causes of dislocation. Apart from the malformation of fracture in the radiographs, the patient should undergo magnetic resonance imaging to assess the annular and quadrate ligaments even the interosseous membrane.

For a simultaneously radial head dislocation and radial shaft fracture, we can perform a closed manipulation to reduce the radial head first, if not succeed, the open reduction and internal fixation of fracture and the radial head would be the next [3, 7–9, 14]. But for a delayed dislocation of the radial head, the more time passed, the more complicated it became, for the space between the radius and ulna begins to fill up with organizing hematoma and fibrosis even after a few days of delay [6]. Jagdeep Singh et al. successfully performed the manual reduction under anesthesia four days after the first operation [13]. But Linzel et al. had to excise the radial head about two months after the fixation of the radial shaft for a non-anatomical reduction of the butterfly fragment that may include the origin of the interosseous membrane [6]. Hiroshi Yamazaki et al. performed the radial osteotomy at the malunion site and repair the annular ligament 6 months after the injury because of an angular deformity of the radial shaft [12]. As for our case, we achieved a good reduction of the butterfly fragment and the alignment, we presumed that the patient may had a dislocation of the radial head but relocated spontaneously preoperatively or at least the annular ligament was disrupted. At the same time, this patient had a BMI index of 36, and we could not sure that he can maintain the forearm in supination with an adjustable brace, which could also influence the repair of the interosseous membrane. After we noticed the dislocation, we tried to close reduce the radial head under fluoroscopy, but this could not be achieved for it had been past for one month, and the patient refused to pursue additional treatment. If accepted, the open reduction of the proximal radioulnar joint, and repair of the annular ligament would be necessary. To avoid this, early diagnosis and treatment are needed. We should carefully examine the proximal radioulnar joint both preoperative and intraoperative, and the magnetic resonance imaging of elbow may be required preoperatively.

To sum up, fracture of the radial shaft is a common injury, but we also need to pay attention to the proximal radioulnar joint, particularly when the fracture occurs in the midshaft to the proximal third of the radial shaft and with a butterfly fragment. The early recognition and appropriate treatment, including good reduction of the butterfly fragment and the alignment, would achieve good results. However, a delayed radial head dislocation would be much more difficult to manage and results in poor clinical outcome.

## Data Availability

The datasets used and/or analysed during the current study are available from the corresponding author on reasonable request.
